# OBESITY MAY UNDERLIE SKELETAL MUSCLE AUTOIMMUNITY IN OLDER ADULTS

**DOI:** 10.1093/geroni/igac059.2455

**Published:** 2022-12-20

**Authors:** Tan Zhang, Xin Feng, Juan Dong, Kristen Beavers, Stephen Kritchevsky, Barbara Nicklas

**Affiliations:** Wake Forest School of Medicine, Winston-Salem, North Carolina, United States; Wake Forest School of Medicine, Winston-Salem, North Carolina, United States; Wake Forest School of Medicine, Winston-Salem, North Carolina, United States; Wake Forest University, Winston-Salem, North Carolina, United States; Wake Forest School of Medicine, Winston-Salem, North Carolina, United States; Wake Forest School of Medicine, Winston-Salem, North Carolina, United States

## Abstract

A mechanistic understanding of the biological basis for sarcopenia could lead to novel biomarkers and intervention targets. We recently reported a novel role of autoimmunity in sarcopenia, which is potentially mediated through cardiac troponin T (cTnT) and its autoantibody. Given the known role of obesity in both autoimmunity and sarcopenia, we further explored the association between circulating cTnT or skeletal muscle IgG and obesity in older adults. We found that, in 37 older adults (NCT01049698, BMI: 30.8 ± 2.3 kg/m²; Age: 69 ± 3.5 yrs) without any cardiac abnormality, serum cTnT was negatively correlated with skeletal muscle volume (r = -0.37, p = 0.02) and leg extension strength (r = -0.38, p = 0.02), but positively correlated with body fat/muscle volume ratio (r = 0.37, p = 0.03). In two other studies including older adults (n = 11 & 12) from a subset of subjects (NCT01049698 & NCT01298817), skeletal muscle IgG1 (GAPDH normalized level by immunoblot) was found to be positively associated with BMI or dual-energy X-ray absorptiometry derived regional fat measurements, including fat mass, abdominal subcutaneous fat, abdominal fat, or thigh fat. We also found that subjects with higher skeletal muscle IgG1 protein levels have significantly higher BMI but lower knee extensor strength. Our finding suggests that obesity may underlie increased circulating cTnT and skeletal muscle IgG infiltration. It also warrants additional investigation into its role in skeletal muscle autoimmunity in older adults.

